# Validation of wearable textile electrodes for ECG monitoring

**DOI:** 10.1007/s00380-019-01347-8

**Published:** 2019-01-24

**Authors:** Yayoi Tetsuou Tsukada, Miwa Tokita, Hiroshige Murata, Yasuhiro Hirasawa, Kenji Yodogawa, Yu-ki Iwasaki, Kuniya Asai, Wataru Shimizu, Nahoko Kasai, Hiroshi Nakashima, Shingo Tsukada

**Affiliations:** 1 Department of Cardiovascular Medicine, Nippon Medical School, 1-1-5 Sendagi, Bunkyo-Ku, Tokyo, 113-8603 Japan; 2 NTT Basic Research Laboratories, NTT Corporation, 3-1 Morinosato Wakamiya Atsugi, Kanagawa, 243-0198 Japan; 3 Hakujikai Memorial Hospital, 5-11-1 Shikahama, Adachi-Ku, Tokyo, 123-0864 Japan

**Keywords:** Textile electrode, Holter monitoring, Wearable device, Washable, Reusable

## Abstract

A highly conductive textile was woven from nano-fibers coated with the PEDOT-PSS polymer. The aim of this study was to assess the usefulness of textile electrodes for ECG recording as a smart garment. Electrode textile pads and lead wires were sewn to the lining of sportswear and their tolerability to repeated washings were tested up to 150 times. The electrical conductivity of the textile electrode remained functional for up to 50 machine washes. To assess the level of motion artifacts or noise during the daily monitoring of ECG, a single lead ECG with conventional or textile electrodes was recorded during supine rest, seated rest, upright trunk rotation (i.e., twisting), and stepping movement in 66 healthy adults. A Holter system was used for data storage and analysis. ECG patterns of P, QRS, and T waves were comparable between the conventional and textile electrodes. However, the signal-to-artifact-and/or-noise ratio (SAR) during twisting was larger in the textile electrodes than in the conventional electrodes. No skin irritation was seen in the textile electrodes. The single lead textile electrodes embedded in an inner garment were usable for continuous and/or repeated ECG monitoring in daily life except during vigorous trunk movement.

## Introduction

ECG recording is one of the most popular cardiac diagnostic tests, because it is noninvasive, inexpensive, simple, and repeatable. Achievements of Holter monitoring in physiology and technology were broad, including various computer-assisted diagnostic methods, long-term recordings, miniaturization of devices, and improved noise-reduction strategies [1–3]. Common problems often raised in Holter monitoring were noncompliance during ECG recording such as intolerance to electrodes due to skin irritation [3] which is caused by gelled electrolytes and/or adhesive tapes needed to fix the electrodes at the same position of the thorax for long-term and/or repetitive recordings [3–5]. There has been a drive to develop dry electrodes since approximately 1965 [6]. Various groups have proposed electrodes without gels or adhesives [7–13]. However, none of these electrodes have achieved routine clinical use for the ECG recording.

Tsukada et al. invented string-shaped electrodes from silk fiber bundles (threads) coated with a conductive polymer, poly (3, 4-ethylenedioxythiophene) (PEDOT)-poly (styrene-sulfonate) (PSS) [14]. This organic string has electro-conductivity, flexibility, and hydrophilicity under both wet and dry conditions; thus, electrolyte paste or solution does not necessarily be needed in recordings of neuronal signal in rat brain. Recently, Tsukada et al. wove a conductive fabric from 700-nm-diameter polyester nano-fibers coated with PEDOT-PSS. This textile electrode is hydrophilic and flexible. These characteristics of this material will allow the nano-fiber textile electrode to adapt gently and mildly to the human skin surface, which are expected to improve ECG quality and stability. Therefore, the aim of our study was (1) to define electrical and physiological characteristics of the textile electrode, (2) to examine the durability of its electrical conductivity after many washes, and (3) to assess ECG waveform in Holter monitoring.

## Materials and methods

### Preparation of wearable electrodes

**Fig. 1:**
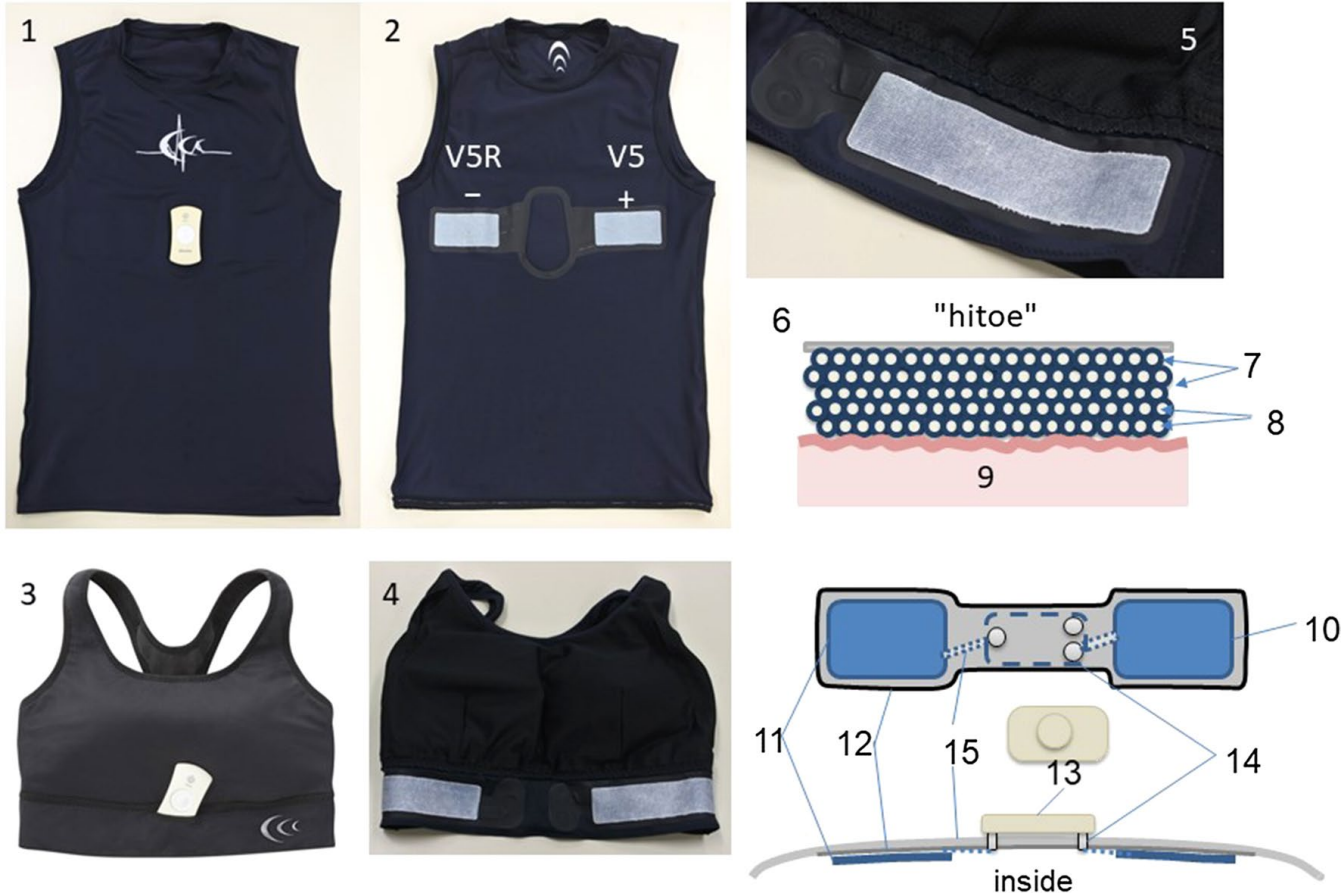
Wearable ECG of smart garment. The ECG sensor patches (5) were placed inside the undershirt (“1. 2.” outside and inside faces for men or the bra strap “3.4.” for women). (6) Cross-sectional view of the textile electrode (hitoe^®^). The cloth of polyester nanofiber (8) was coated by electroconductive polymer PEDOT-PSS (7, blue circles), which surface was directly placed on the skin (9). Back side of the electroconductive textile was fixed on the waterproof layer (12) and the cloth of the underwear (12). ECG signals were conducted through the textile electrodes (5. 10. 11) to electro-conductive yarn (15) and a snap hook button (14); then, these ECG electrodes and the lead wire were connected to the ECG transmitter (13)

The hitoe^®^ (Toray Inc., Tokyo, Japan) electrode was prepared from electroconductive textile fabric which is made of nano-fiber yarn (fiber diameter 700 nm; polyester) coated with PEDOT-PSS polymer thermobonding composition (Fig. 1; 6–7, 8). For assessing the electric property of the electroconductive textile, various types of hitoe^®^ pad (4 cm × 4 cm, 4 cm × 8 cm and 10 cm × 2.8 cm) were cut from PEDOT-PSS-coated textile.

**Fig. 2:**
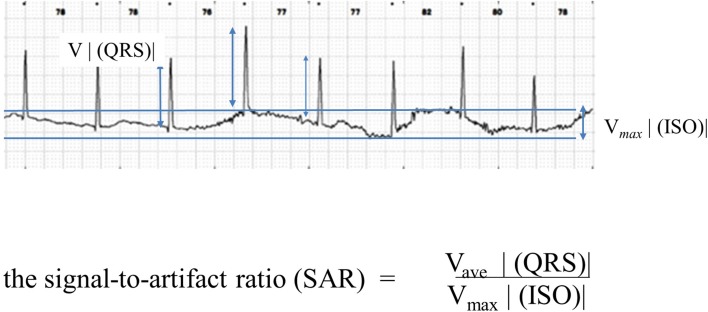
Signal voltage (QRS voltage)-to-artifact voltage ratio (SAR). The ratio of the QRS voltage to a baseline shift was quantified and termed the signal voltage (QRS voltage)-to-artifact voltage ratio or the extent of the drift level of the isoelectric line (SAR). In the present study, the SAR was calculated using a modification of Meziane’s method conditions

**Fig. 3:**
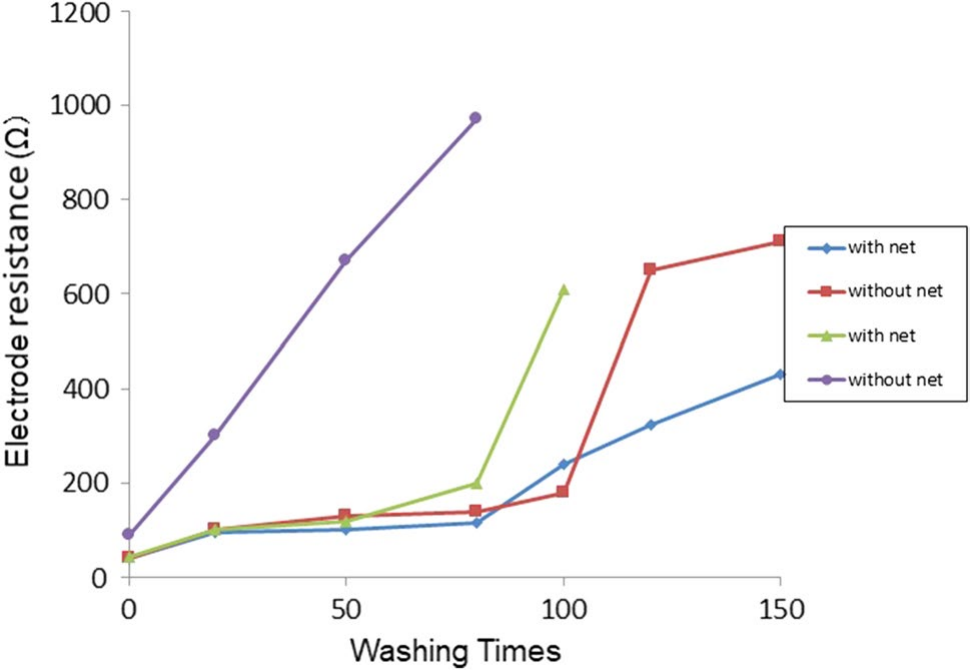
Washing durability of the textile electrodes

**Fig. 4:**
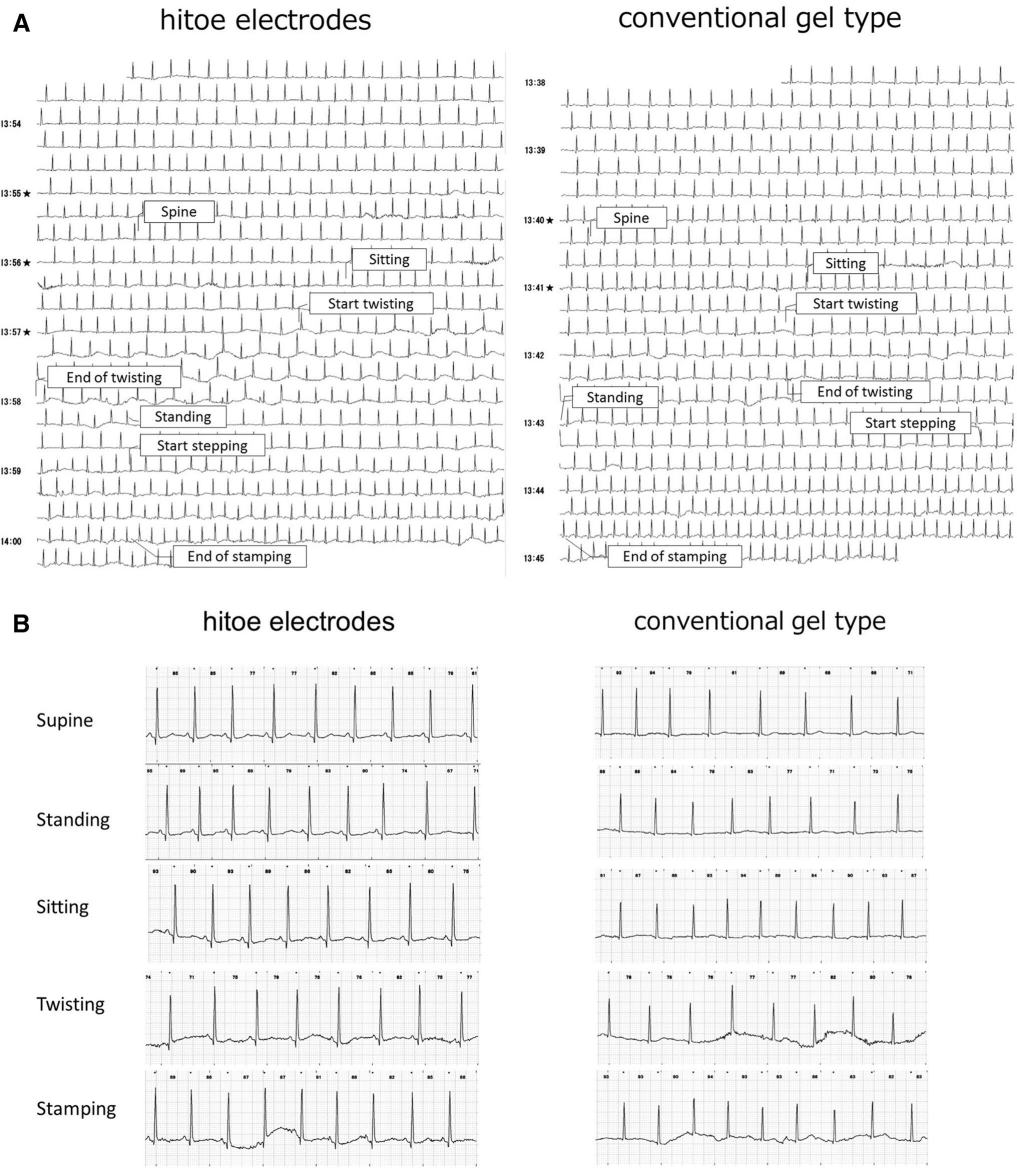
Representative tracings of the textile and conventional gel electrodes

**Fig. 5:**
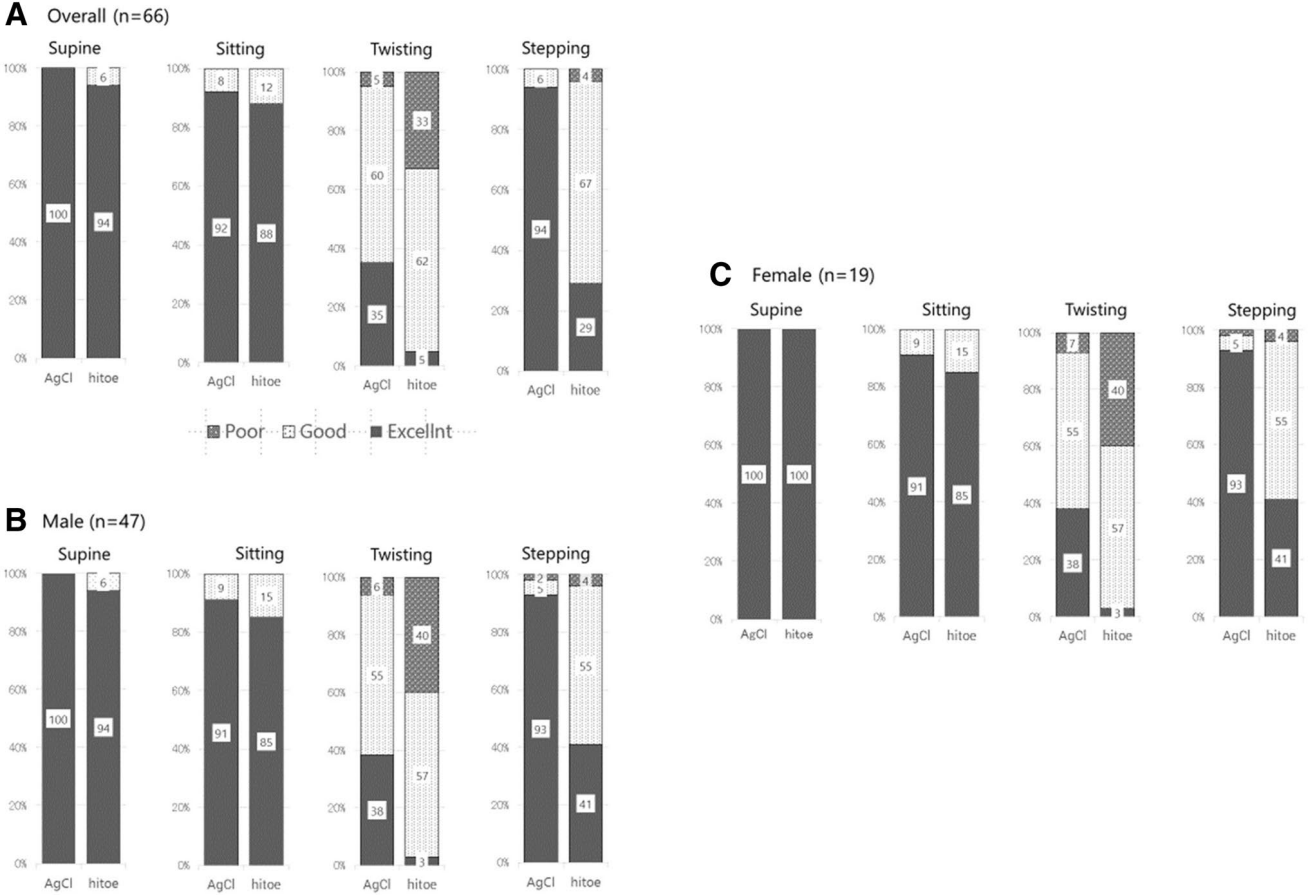
Comparison of waveform recognition between the gel-type conventional electrode and the textile electrode at rest and during postural changes. **a** All volunteer subjects (*n* = 66). **b** Males (*n* = 47). **c** Females (*n* = 19)

Two types of wearable electrodes were sewed (C3fit IN-Pulse^®^, GOLDWIN Inc., Toyama, Japan): T shirts for men and brassiere for women (Fig. 1; 1 and 3). ECG electrodes made of hitoe^®^ patch were sewn to the reverse of the cloth (Fig. 1; 2 and 4). Both the types of wear were compressive to the skin for sticking snugly to the electrodes. The size of electrodes was 8 cm × 4 cm for men’s T shirts and 10 cm × 2.8 cm for women’s brassiere, where the distance between the center of each electrode is 20 cm (Fig. 1; 5). These embedded bipolar electrodes were placed on V5R and V5, which correspond to lead I [15]. An insulated electroconductive lead ribbon was made of coated silver wires which connected between a hitoe^®^ patch and a terminal of ECG device (Fig. 1; 6–14, 15).

### Electrical characteristics of the hitoe^®^ electrode

DC resistance, AC impedance, offset voltage, internal noise, and polarization voltage for 5 s after a defibrillator discharge at 200 V were measured on hitoe^®^ pads in dry condition. DC resistance (sheet resistance), which represents horizontal electroconductivity of the material, was measured by four-point needle electro-conductive meter (Loresta-AX, MCP-T370, Mitsubishi Chemical Analytech, Japan). For AC impedance measurement, a pair of electrodes faced each other and layered over 200 g weight were connected to the bio-electrode impedance meter at a 10-Hz sine wave (Melon Technos, Japan). Offset voltage (twin electrodes were faced to each other) and defibrillator recovery potential were taken by digital multifunctional meter (DM2561A, NF Corporation, Japan). Two hundred voltage loaded to the defibrillator was applied to these electrodes according to the schematics and protocols of ANSI. Bio-signal waveform conformation, electrode’s combined offset instability, and internal noises were measured by polygraph (AP-1124, Miyuki-giken Japan) or neuro-physical recorder (MEB-5504 Nihon Koden Japan), respectively, at adequate frequency range (0.01–1000 Hz). These measurements are mandated for disposal electrodes by the American National Standards Institute (ANSI)/Association for the Advancement of Medical Instrumentation (AAMI) [16].

The electrical contact impedances to the subjects’ skin were measured simultaneously on textile (hitoe^®^ 4 cm × 8 cm two electrodes were fixed inside the underwear, Fig. 1) and conventional gel-type (round shape Ag–AgCl with electrolyte liquid gel, 25 mm in diameter, with adhesive pad 5 cm, Vitrode R, Nihon Koden, Japan) electrodes on 74 volunteers using a bio-electrode impedance meter at a 10-Hz sine wave (Melon Technos, Japan) (protocol 1). Each electrode pad was immersed in 500 μl of a 30% glycerol/0.9% NaCl solution to wet the surface 1 h before use, according to the instruction of the electrodes. Subject’s skin was pretreated with alcoholic wiper for conventional electrodes, but not for textile electrodes.

### Electrical conductivity of textile electrodes in repeated washings

Four smart shirts with textile electrodes were washed repeatedly with (*n* = 2) or without (*n* = 2) a mesh laundry net according to the Japan Industrial Standard (JIS) protocol [17]. Briefly, one washing cycle consisted of 5 min of gentle machine washing at 30 °C (JIS #105) or slightly harder machine washing at 40 °C (JIS #103) using synthetic detergent, followed by two rounds of a machine rinse for 2 min each with clean water and drying at a temperature below 80 °C. This cycle was repeated more than 150 times. Between each machine wash cycle, a 5-min spin-dry procedure was interposed for dehydration. After each washing cycle, the sheet resistance of each pad was measured using the in-line four-point probe technique at dried condition.

### ECG quality during exercise in healthy volunteers (protocol 2)

Forty-seven men and 19 women enrolled to study the ECG signal quality during supine rest, sitting, and physical exercises (Table 1). None of the participants had any history of cardiovascular disease or skin disease. All subjects took their chest measurements to prepare a wearable device fitted tightly to each subject’s thorax. In five subjects, this smart shirt was still loose; therefore, safety pins were used to make the shirt fit more tightly to their thorax.

**Table 1:** Participant characteristics

	Gender		Overall (*N* = 66)
	Male (*n* = 47)	Female (*n* = 19)	
Age (years)	34 ± 10	39 ± 10	35.5 ± 10.3
Height (cm)	171.2 ± 4.8	157.3 ± 5.5	167.2 ± 8.1
BW (kg)	62.1 ± 7.0	52.1 ± 5.4	58.2 ± 8.0
BMI	21.2 ± 1.9	21.0 ± 1.6	21.1 ± 1.8
Chest Circumference (cm)	87.0 ± 4.9	73.5 ± 3.9	82.1 ± 12.7

Values are reported as the mean ± SD

*n* number of participants, *BW* body weight, *BMI* body mass index

In ECG recordings using a Holter ECG recorder (Kenz Cardy 303 Pico+^®^, SUZUKEN Co., Ltd., Nagoya, Japan) (Fig. 1; 6–13), we studied the textile electrodes first. On wearing the smart shirt, the electrode pad was wetted with a few drops of glycerol (C_3_H_8_O_3_). Skin abrader, gel, and adhesive were not applied. ECG recordings were conducted sequentially in a supine position for 30 s, sitting at a comfortable position for 30 s, a trunk rotation (the left and right shoulders are alternately protruded forwardly, just like twisting movement) in a sitting position on the chair for 60 s, and stepping for 90 s. The timing of each twisting or stepping exercise was adjusted to metronomic sounds (1.0 Hz). At the end of the first protocol, the subjects’ skin was observed for any signs of irritation.

Ten minutes after the above protocol, each volunteer’s skin was cleaned with 0.2% chlorhexidine gluconate and abraded gently with a skin preprocessor; then, conventional gel-type electrodes (Cardyrode-P^®^, SUZUKEN) were applied and fixed firmly with gel and adhesive on modified lead I. ECG recordings were repeated on the same four conditions described above. At the end of the protocol, we again observed skin conditions at the sites, where the electrodes were attached.

## Ethical issue

The experimental protocol complies with the Declaration of Helsinki for Human Subjects in Research and was approved by the institutional ethics committee of NTT Basic Research Laboratories (protocol 1) and Nippon Medical School (protocol 2). Informed consent was obtained from all subjects.

## Data analysis

### Qualitative assessments of recorded signals

The recorded whole ECG signals were printed out using the Holter ECG analyzer (Kenz Cardy Analyzer 05^®^, SUZUKEN Co., Ltd., Nagoya, Japan) in 66 volunteers (Protocol 1) to determine the shapes of P, QRS, and T waves and to identify artifacts. Tiny spikes, notches, large baseline swings, a widened isoelectric line, loss of ECG signal, and electromagnetic interference (EMI) [17] were defined as artifacts.

ECG waveforms of P, ORS, and T for each subject were reviewed by two of three certified electrophysiologists (board-certified members of the Japanese Heart Rhythm Society: K.Y., Y.H., and H.M.), and the ECG signal quality was scored in each protocol from 1 to 3 points. Point “3” (a top quality) was assigned for stable recordings lacking motion artifacts, Point “2” for a satisfactory recording with a motion artifact, and point “1” for a poor recording such as with any loss of the ECG waveforms due to a large shift in the isoelectric level and electromyogram (EMG). The scores of the two electrophysiologists were added, and the patients’ ECG quality was categorized as ‘excellent’ for 6 points, ‘good' for 4–5 points, and ‘bad or poor' for 2–3 points. From the consensus of above three cardiologists, ‘excellent’ and ‘good’ are defined as clinically acceptable recordings.

### Signal-to-artifact ratio (SAR)

The ratio of the QRS voltage to a baseline shift was quantified and termed the signal voltage- (QRS voltage)-to-artifact voltage ratio or the extent of the drift level of the isoelectric line (SAR). In the present study, the SAR was calculated using a modification of Meziane’s method (Fig. 2) [9].

### Statistical analysis

All data are expressed as the means ± SD. The data management and statistical analyzes were performed using JMP^®^ (Version 9, SAS Institute Inc.). The differences in average impedances between electrode types were assessed by a two-tailed Student’s *t* test. We used Wilcoxon’s signed rank test for comparison of the SAR and the Chi-square test for evaluation of the ECG quality. A *p* value <0.05 was considered significant.

## Results

### Electrode characteristics and washing durability

The electrode characteristics of the present textile electrodes were accorded well with the ANSI/AAMI guidelines (Table 2). The electrode-skin combined impedances (Protocol 1) were 135 ± 35, and 219 ± 17 kΩ in the textile and conventional electrodes (*n* = 74), respectively (*p* < 0.01).

**Table 2:** Electrode characteristics and ANSI/AAMI requirements for disposable ECG electrodes

	Textile	ANSI/AAMI
DC resistance	< 0.1 KΩ	< 2 KΩ
AC impedance (12)	1.26 ± 0.18 KΩ	< 3 KΩ
Internal noise	1–3 μV	< 150 μV
Defibrillation discharge at 200 V		
Recovery PV	0.195 ± 0.80 mV	< 100 mV
Rate of change of PV	No change	< 1 mV/sec
AC impedance after test	1.14 ± 0.07 KΩ	< 3 KΩ
DC offset voltage (12)	0.0028 ± 0.0020 mV	< 100 mV

Data are reported as the means ± SD

*ANSI* American National Standard Institute, *AAMI* Association for the Advancement of Medical Instrumentation, *ANSI/AAMI* standards: Ref. [14], *PV* depolarizing voltage, *n*: number of experiments

A sharp increase in the electrical resistance of the pads was noted during repeated washings under the slightly hard condition (JIS, #103) without the use of a mesh laundry bag (Fig. 3). Gentle machine-washing (JIS, #105) using a mesh laundry bag was better for maintaining electrical conductivity of the textile shirt than washing without the use of a bag. Up to 50 machine-washing cycles with the use of a mesh laundry bag, electrical conductivity was maintained.

### Signal quality of recorded ECG

As shown in the representative tracings (Fig. 4), P, QRS, and T waves were comparable between the textile and conventional electrodes. No heart beats were missing from any of the ECG recordings of either the textile or conventional electrodes under the supine, sitting, or stepping condition. SAR was better in conventional than textile electrodes and was worst in twist among four situations (Table 3). The quality scores of each waveform during the stepping exercise were excellent in more than 88% of the subjects in either textile or conventional electrode types (Fig. 5).

**Table 3:** Differences in the signal-to-artifact ratio between the textile and conventional electrodes

	Textile (66)	Conventional (66)	*p*
Supine	14.0 ± 8.6	19.5 ± 10.7	0.001
Sitting	12.0 ± 8.0	16.6 ± 8.2	0.001
Twist	2.5 ± 3.0	9.8 ± 6.7	0.001
Stepping	9.3 ± 8.8	13.2 ± 9.3	0.01

Values are reported as the means ± SD

*n* number of participants, including both men and women

High-frequency noise and a baseline shift were noted during twist movements in both the textile and conventional electrodes. The P wave was clearly detectable in supine, sitting, and stepping conditions, as represented in Fig. 4b. In twisting condition, P waves were detected in 17% of cases and others were lost in artifacts.

### Gender difference

SAR was better in men than women in either textile or conventional electrodes and was comparable in supine, sitting, or stepping (Table 4). The twisting motion caused the most deleterious effect on the ECG waveform among four conditions in both textile and conventional electrodes in either men or women. Approximately two-thirds of the recordings were categorized as ‘good’ in the textile group, and the proportions of poor recordings (i.e., QRS and T waves that were not recognizable) were 19% and 33% for conventional and textile electrodes, respectively. Noise was detected in 41% of the recordings of men during twisting with textile electrodes, while that was 6% with conventional electrodes (Fig. 4).

**Table 4:** Differences in the signal-to-artifact ratio between men and women for the textile and conventional gel electrodes

	Men (47)	Women (19)	*p*
Supine			
Textile	14.5 ± 9.5	9.8 ± 4.5	0.05
Conventional	23.4 ± 10.0	12.6 ± 6.0	0.0001
Sitting			
Textile	11.5 ± 8.7	9.6 ± 5.3	NS
Conventional	18.5 ± 7.9	11.5 ± 6.7	0.001
Twist			
Textile	2.4 ± 3.0	2.9 ± 5.3	NS
Conventional	11.3 ± 7.2	5.7 ± 2.4	0.001
Stepping			
Textile	11.3 ± 7.2	4.2 ± 5.9	0.01
Conventional	16.2 ± 9.3	5.7 ± 2.4	0.0001

Values are reported as the means ± SD

*n* number of participants

## Discussion

The present study showed the usefulness and limitations of a smart garment or a wearable and washable shirt for ECG monitoring. The advantages of the present smart garment were (1) simply wearing the shirt with a single lead of electrodes will minimize the technicians’ involvement, (2) ECG monitoring at home care will be feasible due to the prefixed electrode position and wiring connection, and (3) continuous long-lasting ECG monitoring will be allowable due to the absence of electrode-related skin injuries. Electrode and lead misplacement is a frequent pitfall of ECG monitoring [18, 19], where changes in ECG morphology were occasionally interpreted as ischemic in origin or as serious arrhythmias [20]. In a real clinical setting, it is sometimes required to displace the electrode position on patient’s chest wall to avoid skin erosion. The present smart garment was free of gel and adhesive, which should avoid skin irritation or injury.

The electrical characteristics of the present textile pad fulfilled all of the standards required for commercial disposable electrodes [16]. Since repetitive gentle washings using a mesh laundry bag did not impair their electrical conductivity for at least 50 runs (Fig. 2), the present textile electrodes would be usable for repeated ECG measurements at the same fixed electrode positions.

A critical level of sheet resistance is less than 200 Ω, which corresponded to the electroconductivity of the polymer (PEDOT-PSS) per se. The present textile electrodes of hitoe^®^ maintained the level of the electric conductivity of PEDOT-PSS, which will prove one of advantages in using nano-fibers. Usability of PEDOT-PSS was recently extended to printed electrodes [21]; however, its washing durability was not reported.

ECG signal impairment was often observed when the skin became desiccated. In the present study, the sheet resistance exceeded the level of more than 500 Ω in the absence of an electrolyte solution pretreatment to the textile electrodes. The contact impedance, which originated from the attachment of a pair of electrodes to the body surface, should be low for the hitoe^®^ textile electrodes as for the conventional gel electrodes. The low level of contact impedance may be necessary to minimize the extent of baseline shifts or internal noise. The advantages of ECG signal quality in textile electrodes compared with conventional gel electrodes were previously reported in the resting and exercise condition [22].

In the present study, we tested the effects of daily body motions on the ECG configuration and its isoelectric level using textile and conventional gel-type electrodes. The latter type was a commercial device designed for one-time use. We confirmed that the ECG traces of the wearable electrodes were very comparable to those of the conventional gel-type electrodes not only in the supine and sitting positions but also during the stepping exercise. However, the SAR deteriorated more during the twisting movement with the textile electrodes than with the conventional electrodes, which might be related to the skin deformation and skin-electrode reciprocal movement, such as experienced during twisting. The commercial device was more robust to motion artifacts than the textile electrode, which was one of obvious disadvantages of the textile electrodes.

As presented in Tables 3, 4, significantly low level of SAR was derived from insufficient electrode-skin contact. The traction of the underwear’s cloth during rapid twist movements made the electrodes floating or deformity, which may be one of main reasons of the baseline shift. The rapid, sharp, and low-amplitude signals that were similarly observed with the textile and conventional gel electrodes also during twist movements, indicating the presence of EMG from skeletal muscles. These motion artifacts should be resolved in a future study.

Clinical experience with Holter ECG recordings has suggested that gender differences in body motion affect ECG stability due to breast motion, or differences in the shape and size of the torso, and the strength of thoracic muscles. Accordingly, we prepared different types of smart shirts between men and women. Nevertheless, the ECG quality was still better in men than in women.

In conclusion, the present conductive, soft, and flexible textile was usable as an ECG pad for long-term monitoring. Since the present electrodes were washable, reusable and free of gel and adhesive, the cost related to manufacturing the fabric textile coated with PEDOT-PSS will be minimized. The present findings also suggest that the uses of ECG monitoring will extend from the classical clinical applications of detecting arrhythmias or ischemic changes to real-time practice through ongoing ECG monitoring, sports medicine for higher performance, safety management in risky circumstances, such as heated workplaces, or home health care through adding telemetry or ICT [23–25].
